# Establishing the best combination of the kappa free light chain index and oligoclonal bands for an accurate diagnosis of multiple sclerosis

**DOI:** 10.3389/fimmu.2023.1288169

**Published:** 2023-10-25

**Authors:** Enric Monreal, José Ignacio Fernández-Velasco, Ana García-Soidán, Susana Sainz de la Maza, Mercedes Espiño, Noelia Villarrubia, Fernando Rodríguez-Jorge, Juan Luís Chico-García, Raquel Sainz-Amo, Jaime Masjuan, Lucienne Costa-Frossard, Luisa María Villar

**Affiliations:** ^1^ Department of Neurology, Hospital Universitario Ramón y Cajal, Red Española de Esclerosis Múltiple (REEM), Instituto Ramón y Cajal de Investigación Sanitaria (IRYCIS), Universidad de Alcalá, Madrid, Spain; ^2^ Department of Immunology, Hospital Universitario Ramón y Cajal, Red Española de Esclerosis Múltiple (REEM), Instituto Ramón y Cajal de Investigación Sanitaria (IRYCIS), Universidad de Alcalá, Madrid, Spain

**Keywords:** clinically isolated syndrome, multiple sclerosis, kappa free light chain, oligoclonal bands, diagnosis

## Abstract

**Introduction:**

The immunoglobulin kappa free light chain (KFLC) index has been proposed as a potentially suitable alternative to oligoclonal IgG bands (OCGB) for diagnosing multiple sclerosis (MS), offering automation and reduced processing time. However, there is no consensus on the preferred approach or how to combine both techniques.

**Methods:**

This prospective cohort study aimed to determine the best utilization of OCGB and KFLC index in patients with a clinically isolated syndrome (CIS) followed for at least two years. OCGB and KFLC were assessed using isoelectric focusing and immunoblotting and turbidimetry, respectively. Sensitivity, specificity, and accuracy for diagnosing MS were calculated for each method.

**Results:**

The study included 371 patients, with 260 (70.1 %) being women, and a median age of 34.9 (27.8 – 43.9) years. Using a cut-off value of 6.1, the KFLC index demonstrated a sensitivity and specificity of 86.3% and 93.9%, respectively. The sensitivity of OCGB (95.3%) was higher (p < 0.001 vs. KFLC index) and the specificity (100%) was comparable to that of the KFLC index (p = 0.5). The concordance between the methods was not uniform across all patients, with 97.8% agreement in patients with KFLC index ≥ 6.1 and 56.0 % in patients with KFLC index < 6.1. In patients with a KFLC index < 6.1, OCGB still identified 75.0 % of MS patients due to its higher sensitivity. An algorithm using the KFLC index as a screening tool and OCGB as an alternative for patients with a negative KFLC index result achieved an accuracy of 96.3 %.

**Discussion:**

Combining the KFLC index and OCGB can provide an easily reproducible and accurate method for diagnosing MS, with OCGB primarily reserved for patients with a KFLC index < 6.1.

## 1 Introduction

The diagnosis of multiple sclerosis (MS) lacks a pathognomonic test and has traditionally relied on clinical and radiological findings demonstrating a dissemination in space (DIS) and time (DIT) not attributable to any other disease. Paraclinical tests, including magnetic resonance imaging (MRI) and intrathecal IgG synthesis (ITGS), have been shown to expedite the diagnosis, as outlined in the 2017 revised McDonald criteria (1). ITGS is a hallmark of MS and is present in >95% of patients. It can be demonstrated in paired cerebrospinal fluid (CSF) and serum samples through semiquantitative or qualitative tests (oligoclonal IgG bands [OCGBs]), with the latter considered the gold standard (2). OCGBs demonstrate the inflammatory nature of the symptoms and allow for the early diagnosis of MS in cases in which DIT cannot be demonstrated (1). OCGBs have exhibited high reproducibility for the detection of ITGS (3), and the utilization of commercial kits has simplified their detection. However, their performance remains laborious, and experienced labs are required for accurate interpretation. On the other hand, several recent studies have evaluated the diagnostic value of kappa free light chains (KFLCs) as an alternative method for detecting ITGS (4–6). The KFLC index can be detected using automated methods, reducing time consumption and eliminating the need for experienced labs for detection (7), thus improving inter-rater results (5, 8–10). Nevertheless, various methods of analysis and different cutoffs have been described, limiting its widespread use in clinical practice. A recent meta-analysis comprehensively analyzed the sensitivity and specificity of the KFLC index and OCGBs from all previous studies, showing no significant differences between them in discriminating MS, irrespective of the method and assay used for KFLC quantification (6). However, determining the optimal cutoff value and the feasibility of combining the KFLC index with OCGBs, as well as identifying the samples for which this second test should be evaluated, are necessary before implementing the KFLC index in clinical practice (11–13).

Our objective was to compare the diagnostic value of the KFLC index and OCGBs in patients with a clinically isolated syndrome (CIS) suggestive of MS and propose an algorithm for diagnosing MS using both techniques.

## 2 Materials and methods

### 2.1 Study design

This was a single-center observational study conducted at the Hospital Universitario Ramón y Cajal (HRYC) referral MS center, Madrid. The study followed the Strengthening the Reporting of Observational Studies in Epidemiology (STROBE) reporting guideline. We enrolled consecutive patients with a clinically isolated syndrome (CIS) and prospectively collected data meeting the following inclusion criteria: a baseline magnetic resonance imaging (MRI) within 6 months of disease onset and stored serum/CSF samples available for analysis obtained at our center. All patients provided their signed informed consent prior to inclusion. The exclusion criteria were as follows: 1) history of confirmed or possible previous relapses before the first visit; 2) lumbar puncture (LP) performed during corticosteroid treatment or under disease-modifying treatments (DMTs); 3) follow-up of less than 2 years (unless a diagnosis of MS was confirmed earlier); and 4) incomplete CSF data. The prospective follow-up involved assessing new possible relapses and Expanded Disability Status Scale (EDSS) examinations by certified neurologists at least every 6 months. Additionally, brain MRI was conducted at least annually.

### 2.2 Ethical approval

The study was approved by the institutional ethics board of HRYC. A signed informed consent was obtained from all patients.

### 2.3 Data collection

All patients meeting the eligibility criteria from June 1, 1994, to November 24, 2021, were included in this study, with the follow-up period extending until February 1, 2023. The collected variables encompassed demographic, clinical, radiological, and CSF data. The use of DMTs along with the dates of initiation and discontinuation were also meticulously recorded. Disability assessment, based on the EDSS, was conducted at first relapse, at least every 6 months thereafter, and additionally in the case of new relapses. MRI studies were performed at baseline using a 1.5T or 3T magnet, capturing a range of sequences, including transverse spin-echo proton-density weighted and/or T2-weighted spin-echo, as well as the transverse and sagittal T2-fluid-attenuated inversion recovery sequence. Brain scans also encompassed transverse T1-weighted spin-echo before and after contrast injection. The obtained spinal cord sequences included T2-weighted fast spin-echo and sagittal short-tau inversion recovery. Gadolinium-enhanced sagittal T1-weighted and axial T2-weighted fast spin-echo were performed, covering segments with confirmed or suspected lesions identified in the sagittal sequences. Lumbar punctures were conducted by trained neurologists before the administration of corticosteroids and DMT. CIS was defined as the first clinical episode, presenting patient-reported symptoms and objective findings indicative of an inflammatory demyelinating event in the central nervous system. These episodes had a duration of at least 24 h and occurred in the absence of fever or infection. The 2017 revised McDonald criteria were applied for the diagnosis of CIS or MS (1).

### 2.4 CSF analysis

Paired serum and CSF samples were obtained and stored at –80°C until they were assayed. Serum and CSF IgM, IgG, and albumin levels were quantified using nephelometry with a BN ProSpec nephelometer (Siemens Healthcare Diagnostics, Marburg, Germany). OCGBs were analyzed in serum and CSF using isoelectric focusing (IEF) and immunoblotting as previously described (14). OCGBs were deemed positive if two or more IgG bands were detected in the CSF but not in the paired serum sample. KFLCs were analyzed by turbidimetry using Optilite Freelite Mx Kappa FreeKits, and the analysis was performed in an Optilite turbidimeter (Binding site, Birmingham. UK). The KFLC index was calculated using the formula: Q_KFLC_/Q_albumin_, where Q_KFLC_ and Q_albumin_ represent the CSF/serum quotients of KFLCs and albumin, respectively. In cases in which CSF KFLCs were undetectable, we established the value of the lower detection limit (0.27 mg/l). In these cases, we excluded those patients in whom the KFLC index yielded values higher than 6.1 due to the serum KFLC or the Q_albumin_ levels. The cutoffs used for the KFLC index were ≥6.1 based on the value with the maximum Youden index in our cohort and previous studies (6), and ≥6.6 (4) based on literature.

### 2.5 Statistical analysis

Categorical variables were described using absolute and relative frequencies, while continuous variables were presented as medians with interquartile ranges (IQRs). Baseline characteristics were analyzed with parametric and non-parametric tests as appropriate. Cohen’s kappa statistic was used to assess the between-methods agreement. The area under the curve (AUC) was calculated using a receiver operating characteristic (ROC) curve to determine the optimal KFLC index cutoff, defined as the cutoff yielding the maximum Youden index. We analyzed sensitivity, specificity, and accuracy for all KFLC index cutoffs along with corresponding 95% confidence interval (CI) values. Additionally, these indices were calculated for IgG OCBs in the total cohort and among patients with a KFLC index below and above 6.1. Sensitivity, specificity, and accuracy were computed using the following formulas:


$$•Sensitivity:\left(TP/\left[TP+FN\right]\right)\times 100$$

$$•Specificity:\left(TN/\left[TN+FP\right]\right)\times 100$$

$$•Accuracy:\left(\left[TP+TN\right]/\left[TP+TN+FP+FN\right]\right)\times 100$$

True positives (TPs) were defined as positive results for the KFLC index or OCGBs that were subsequently diagnosed as MS. True negatives (TNs) were defined as CIS patients with a negative KFLC index or OCGB result. False positives (FPs) were defined as patients with CIS but yielding positive results for the KFLC index/OCGBs, whereas false negatives (FNs) were defined as patients with MS but negative KFLC index/OCGB results. The McNemar test was employed to compare sensitivity and specificity between the methods. All analyses were performed using Stata 17 (StataCorp, College Station, TX, USA). All tests were two tailed, and p< 0.05 was considered significant.

## 3 Results

### 3.1 Descriptive analysis

**Figure 1:**
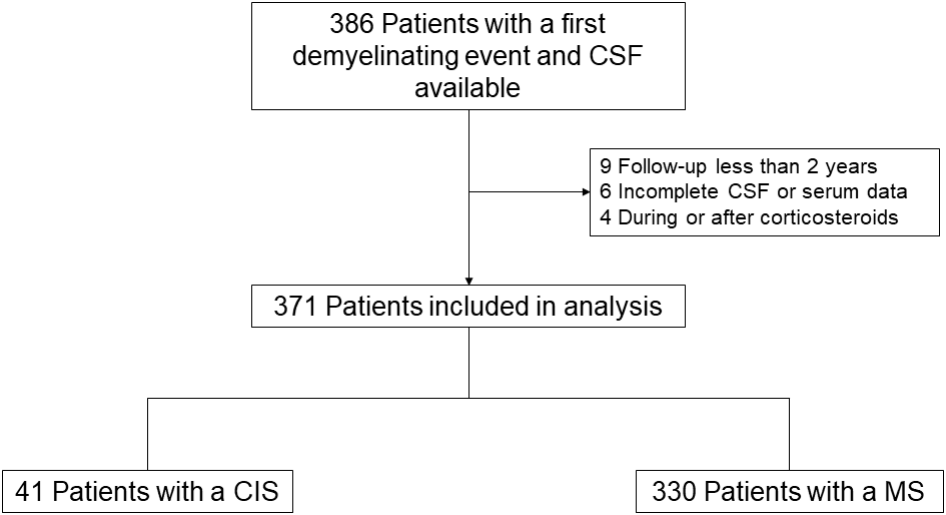
Flowchart of participants. CIS, clinically isolated syndrome; CSF, cerebrospinal fluid; MS, multiple sclerosis.

A total of 386 patients with CIS and available serum/CSF samples were initially selected. After excluding nine patients with less than 2 years of follow-up, six patients with incomplete data, and four patients who received corticosteroids during or after LP, 371 patients were included in the analysis (**Figure 1**). Among these, 330 (89%) patients fulfilled the 2017 revised McDonald criteria for MS after a median follow-up of 6.23 (IQR 3.85–10.2) years, while 41 (11%) continued to have CIS. Only 134 (36.1%) patients were followed up for less than 5 years, and undetectable levels of CSF KFLCs were observed in 52 (14.0%) of patients. We further excluded from statistical analyses those patients who exhibited undetectable CSF KFLCs and high KFLC index values (n = 18, which represents a 4.9% from the total cohort). Baseline characteristics of the cohort are presented in **Table 1**, with a median (IQR) age at baseline (considered as time of LP) of 34.9 (27.8–43.9) years. There were 260 women (70.1%) in the cohort.

**Table 1:** Patient characteristics.

	Total (n = 371)	CIS patients (n = 41)	MS patients (n = 330)	P value
Female	260 (70.1)	28 (68.3)	232 (70.3)	0.79
Age (years)	34.9 (27.8 – 43.9)	41.2 (33.8 – 48.6)	34.2 (27.6 – 43.4)	0.002
Time to LP after first relapse (months)	4.33 (1.38 – 9.08)	6.53 (3.48 – 15.5)	4.08 (1.21 – 8.66)	0.09
Topography of first relapse				
Optic nerve	80 (21.6)	17 (41.5)	63 (19.1)	0.005
Brainstem	80 (21.6)	5 (12.2)	75 (22.7)	
Spinal cord	147 (39.6)	9 (22.0)	138 (41.8)	
Cerebral hemisphere	49 (13.2)	8 (19.5)	41 (12.4)	
Multifocal	10 (2.7)	2 (4.9)	8 (2.4)	
Paroxysmal symptoms	5 (1.4)	0	5 (1.5)	
DIS and DIT criteria at baseline MRI				
Neither DIS or DIT	59 (15.9)	37 (90.2)	22 (6.7)	< 0.001
DIS without DIT	139 (37.5)	4 (9.8)	135 (40.9)	
DIS and DIT	173 (46.6)	0	173 (52.4)	
EDSS score at baseline	1.5 (1 – 2)	2 (1.5 – 2)	1.5 (1 – 2)	0.02
T2 lesions at baseline				
0	24 (6.5)	13 (31.7)	11 (3.3)	< 0.001
1 – 3	56 (15.1)	14 (34.2)	42 (12.7)	
4 – 9	98 (26.4)	4 (9.8)	94 (28.5)	
≥10	193 (52.0)	10 (24.4)	183 (55.5)	
Patients with enhancing lesions	171/328 (52.1)	5/39 (12.8)	167 (57.8)	< 0.001
CSF data				
IgG oligoclonal bands	311 (83.8)	0	311 (94.2)	< 0.001
IgG index	0.77 (0.60 – 1.09)	0.53 (0.47 – 0.61)	0.82 (0.64 – 1.16)	< 0.001
CSF KFLC levels (mg/dl)	1.88 (0.5 – 6.39)	0.27 (0.27 – 0.31)	2.29 (0.77 – 7.32)	< 0.001
Serum KFLC levels (mg/dl)	14.4 (12.1 – 17.4)	13.5 (10.9 – 15.5)	14.5 (12.2 – 17.5)	0.06
KFLC index	25.7 (5.82 – 75.1)	4.57 (3.14 – 5.00)	32.2 (10.8 – 84.3)	0.002
KFLC ≥5.5	282 (76)	2 (4.9)	280 (84.9)	< 0.001
KFLC ≥6.1	278 (74.9)	2 (4.9)	276 (83.6)	< 0.001
KFLC ≥6.6	276 (74.4)	2 (4.9)	274 (83.0)	< 0.001
DMT use during follow-up^a^	264 (71.2)			
Untreated	107 (28.8)	41 (100)	68 (27.3)	< 0.001
Platform or oral DMTs^b^	221 (59.6)	0	221 (67.0)	< 0.001
Monoclonal antibodies^c^	81 (21.8)	0	81 (24.6)	< 0.001
Time of follow-up (years)	6.23 (3.85 – 10.2)	5.71 (3.50 – 7.81)	6.49 (4.03 – 10.7)	0.04

CSF, cerebrospinal fluid; DMTs, disease-modifying treatments; EDSS, expanded disability status scale; IgG, immunoglobulin G; KFLC, kappa free light chain; LP, lumbar puncture; MRI, magnetic resonance imaging.

Categorical variables are shown as number (%) and differences between groups were tested using χ² or a Fisher exact test, as appropriate. Continuous variables are described as median (interquartile range) and we used Student’s T-test or a Mann–Whitney test, as appropriate.

a Patients might have received different types of DMTs during follow-up.

b Platform or oral DMTs: subcutaneous or intramuscular interferon-ß, glatiramer acetate, teriflunomide, dimethyl fumarate, fingolimod, oral cladribine, daclizumab, azathioprine, tacrolimus.

c Monoclonal antibodies: natalizumab, alemtuzumab, ocrelizumab, rituximab, ofatumumab.

### 3.2 Diagnostic value of the KFLC index and OCGBs

We performed an ROC curve analysis to determine the optimal cutoff value for the KFLC index to discriminate between MS and CIS in our cohort (**Figure e-1**). The KFLC index showed high accuracy in discriminating patients with a final diagnosis of MS (AUC = 0.92, 95% CI 0.89–0.95). Based on the Youden index, the optimal cutoff value was 6.1 (**Figure e-2**), which had the highest sensitivity and specificity. Among the total cohort, 305 (86.4%) patients showed a positive result for OCGBs, and 278 (78.8%) and 276 (78.2%) patients had a KFLC index ≥6.1 and ≥6.6, respectively (**Table 1**). The sensitivity, specificity, and accuracy of these cutoffs to identify patients with MS are shown in **Table 2**. Conversely, the diagnostic properties of OCGBs for a diagnosis of MS were as follows: a sensitivity of 95.3% (95% CI 93.1–97.5), a specificity of 100% (one-sided 97.5% CI 89.4), and an accuracy of 95.8% (95% CI 93.1–97.4). Using the McNemar test, we observed that the sensitivity of OCGBs was significantly higher than the KFLC index independent of the cutoffs used (p< 0.001), but the specificity was similar.

**Table 2:** Diagnostic properties of different KFLC cutoff values and IgG OCBs.

	Sensitivity	Specificity	Accuracy
IgG OCBs	95.3 (93.1 – 97.5)	100 (89.4)^a^	95.8 (93.1 – 97.4)
KFLC index 6.1	86.3^b^ (82.7 – 89.8)	93.9^c^ (91.5 – 96.4)	87.0^b^ (83.0 – 90.1)
KFLC index 6.6	85.6^b^ (82.0 – 89.3)	93.9^c^ (91.5 – 96.4)	86.4^b^ (82.4 – 89.6)

IgG, immunoglobulin G; KFLC, kappa free light chain; OCBs, oligoclonal bands.

a One-sided 97.5% CI.

b P< 0.001 for comparison with IgG OCBs.

c P > 0.05 for comparison with IgG OCBs.

Given their comparable diagnostic performance in terms of the KFLC index cutoff results, we opted for the cutoff value of 6.1, identified as the most discriminatory level in our cohort and in a comprehensive meta-analysis of previous studies (6). First, we investigated whether patients with multiple sclerosis (n = 320) exhibited distinct baseline characteristic based on a KFLC index ≥6.1 versus<6.1. Data are summarized in **e-Table 1**. Notably, we found no significant differences between both groups except for the proportion of patients who had received DMTs during their follow-up. Second, we conducted a comparative analysis of the KFLC index and OCGBs for MS across three groups based on baseline MRI findings: 1) patients who neither fulfilled DIS nor DIT criteria; 2) patients with DIS but not DIT; and 3) patients meeting both DIS and DIT criteria (**e-Table 2**). In the second group (DIS without DIT criteria), OCGBs facilitated the diagnosis of MS in 125/134 (93.3%) of patients, whereas a KFLC index ≥6.1 identified MS in 112/134 (83.6%) of patients (P = 0.002). However, the time to MS diagnosis was comparable between patients with OCGBs (median [IQR] of 4.42 [2.0–10.5] months) and those with a KFLC index ≥6.1 (5.48 [2.20–12.6] months) in this cohort (P = 0.69).

The between-methods agreement of a KFLC index of 6.1 with OCGBs was 89.0%, corresponding to a kappa statistic of 0.62 (substantial agreement) (**Table 3**). Among patients with a KFLC index ≥6.1, the agreement with OCGBs was almost perfect (97.8%), allowing the identification of four (1.44%) more patients with MS than OCGBs; only two (0.72%) patients were classified as FPs. However, in cases in which the KFLC index was<6.1 (n = 75, 21.3%), we observed a substantial increase in the FN results (n = 44, 58.7%), and the agreement with OCGBs was reduced to 56.0%. Among these patients, OCGBs still identified 33/44 (75.0%) patients with MS, showing a sensitivity of 75.0% (95% CI 65.2–84.8). The specificity of OCGBs among patients with a KFLC index<6.1 remained (100%, with a one-sided 97.5% CI of 88.8) (**Table 3**).

**Table 3:** Diagnostic performance of OCBs stratified by the KFLC index cutoff value of 6.1.

	TPs	FPs	FNs	TNs	Sensitivity (95% CI)	Specificity (95% CI)	Agreement	Cohen’s K statistic
Total analyzed (n = 353)								
KFLC index 6.1	276 (78.2%)	2 (0.57%)	44 (12.5%)	31 (8.78%)	86.3 (82.7 – 89.8)	93.9 (91.5 – 96.4)	89.0%	0.62
IgG OCBs	305 (86.4%)	0	15 (4.25%)	33 (9.35%)	95.3 (93.1 – 97.5)	100 (89.4)^a^		
KFLC ≥6.1 (n = 278)								
KFLC 6.1	276 (99.3%)	2 (0.72%)	0	0	-	-	97.8%	-
IgG OCBs	272 (97.8%)	0	4 (1.44%)	2 (0.72%)	98.6 (97.2 – 99.9)	100		
KFLC<6.1 (n = 75)								
KFLC 6.1	0	0	44 (58.7%)	31 (41.3%)	-	-	56.0%	-
IgG OCBs	33 (44.0%)	0	11 (14.7%)	31 (41.3%)	75.0 (65.2 – 84.8)	100 (88.8)^a^		

CI, confidence interval; FNs, false negatives; FPs, false positives; IgG, immunoglobin G; KFLC, kappa free light chain; OCBs, oligoclonal bands; TNs, true negatives; TPs, true positives.

a One-sided 97.5% CI.

### 3.3 Algorithm for the combination of the KFL index and OCGBs

**Figure 2:**
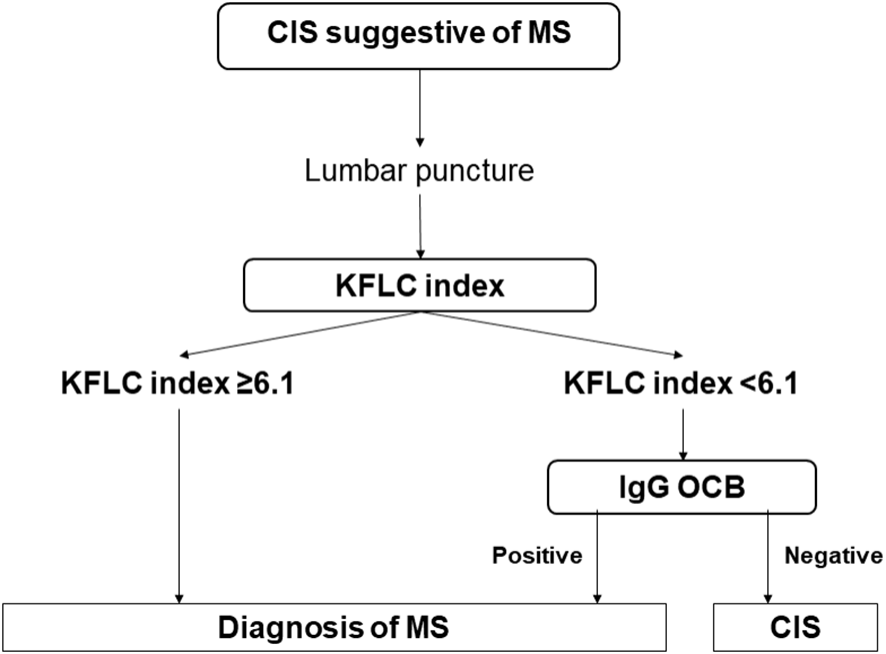
Diagnostic algorithm for patients with a CIS suggestive of MS. The diagnostic algorithm for patients with a CIS suggestive of MS involves first assessing the KFLC index. If a negative result is obtained, performing OCGBs can still identify 72.2% of patients with MS. This algorithm yielded an accuracy of 95% (95% CI 92.7–97.1). CI, confidence interval; CIS, clinically isolated syndrome; IgG, immunoglobulin G; KFLC, kappa free light chain; MS, multiple sclerosis; OCBs, oligoclonal bands.

Based on the diagnostic performance of both techniques to discriminate MS, we propose a diagnostic algorithm to combine the KFLC index and OCGBs in patients with a typical CIS suggestive of MS (**Figure 2**). We recommend first performing a KFLC analysis. If the KFLC index is equal to or above 6.1, OCGBs are not required because the specificity of KFLCs and the between-methods agreement of KFLCs and OCGBs were extremely high. In these cases, the diagnosis of MS could be established. Conversely, if the KFLC index results are below 6.1, OCGBs should be performed due to the non-negligible rate of false negatives obtained with this method. Among these patients, the specificity of OCGBs remained, and thus the diagnosis of MS could also be established. If both the KFLC index and OCGBs are negative, clinical, and radiological follow-up is required as 11/42 (26.2%) of patients were still diagnosed with MS in our cohort. The combination of both techniques in this algorithm yielded a sensitivity of 96.6% (95% CI 94.7–98.5) and a specificity of 93.9% (95% CI 91.5–96.4) for diagnosing MS, which corresponded to an accuracy of 96.3% (95% CI 93.7–97.9) (AUC = 0.95, 95% CI 0.92–0.99).

## 4 Discussion

In this observational study, we included a large cohort of patients with a CIS, followed for a median of 6.2 years, to compare the diagnostic properties of OCGBs and different KFLC index cutoffs for diagnosing MS. The key findings of this study are as follows: 1) the sensitivity and specificity of all KFLC index cutoffs analyzed (with values of approximately 6) did not differ significantly; 2) the specificity of the KFLC index and OCGBs was very high and similar, but the sensitivity of OCGBs was clearly higher than that of the KFLC index; and 3) OCGB analysis should be performed in patients with a negative KFLC index result to still identify patients with MS.

The development of new tests that facilitate ITGS detection is important for MS diagnosis as OCGBs, the gold standard technique, still requires experienced laboratories for its interpretation, leading to differences in sensitivity and specificity between groups (6). In this regard, the value of KFLC detection has arisen as a highly promising candidate. Measuring CSF KFLC concentration or the KFLC index using automated methods provides more uniform results than OCGBs (8–10). The automated methods available for FLC quantification include nephelometry and turbidimetry (15, 16). The sensitivity of both methods is similar, and no differences have observed between the N Latex FLC assay kit (Siemens Healthcare Diagnostics Products GmbH, Marburg, Germany) and the Freelite (The Binding Site Ltd., Birmingham, UK) assay (6, 16, 17). Regarding KFLC measurements, both CSF KFLC concentrations (18) and the KFLC index (4) seem to give good results to predict MS diagnosis in patients with a CIS, although more studies about the KFLC index have been identified in the literature, making this method more consistent (6). However, its specificity may decrease when addressing the differential diagnosis of MS with other inflammatory neurological diseases as they may also have high KFLC index values (17, 19), which occurs less frequently with OCGBs (20). On the other hand, the KFLC index may also be useful at discriminating between non-inflammatory and other inflammatory neurological diseases different from MS (19, 21).

In the context of patients with a CIS, the literature comparing the ability of the KFLC index and OCGBs to detect MS showed heterogeneous results. In some studies, both methods had similar diagnostic properties (10, 22–27) but others described more heterogeneous results (4, 7, 11, 12, 28–32). The discrepancies across studies might be explained by the time of follow-up of patients, the management of cases with undetectable KFLC concentrations, the cutoff used for the KFLC index, and the experience of the lab performing OCGB analysis. When all studies were combined and meta-analyzed, no differences were observed in either sensitivity or specificity between them (6). In our cohort, we found a lower sensitivity of the KFLC index (around 86%) than OCGBs (95.3%) independent of the cutoff evaluated. The specificity was high with both the KFLC index (93.9%) and OCGBs (100%), similar to results obtained in other cohorts (15, 26, 33–35). However, the most interesting results were obtained when we compared the correlation of both methods in patients with positive and negative values for the KFLC index. Although it was nearly perfect for positive cases (97.8%), it decreased to 56.0% in negative ones due to a higher sensitivity of OCGBs. This is interesting as it indicates that positive KFLC index values could substitute OCGBs, thus avoiding a complex and time-consuming technique in most cases (78.8% in our cohort). By contrast, we can increase the sensitivity of the KFLC index by using OCGBs in cases in which the KFLC index yielded a negative result.

These data indicate that the KFLC index can be an optimal method for screening analysis of ITGS, and OCGB can still detect a number of patients with MS with a negative KFLC index result. We suggest combining both methods in an algorithm that showed a high sensitivity (96.6%) and specificity (93.9%), which may reduce costs and time while maintaining the diagnostic accuracy.

Previous studies have also proposed an algorithm for the diagnosis of MS by combining the KFLC index and other methods to detect ITGS, particularly OCGBs (7, 11–13, 36). Interestingly, while there is an agreement on using the KFLC index as a screening method, the aforementioned studies suggested performing OCGB testing in patients with a first positive result, which differed from our recommendation to study patients with a negative KFLC index value. This difference is likely based on their results with the KFLC index, showing high sensitivity but lower specificity, whereas we found higher specificity than sensitivity.

This study has certain limitations. First, the level of detection of the turbidimeter used was 0.27 mg/l, which is higher than in other kits with greater sensitivity that were not available for this study. Although this limitation has been addressed differently in various studies (7, 31, 34), we followed the latest recommendations (6) and set the limit of detection for patients with non-detectable levels of CSF KFLCs. To mitigate bias, we excluded patients with undetectable CSF KFLC concentrations for whom the KFLC index yielded high values (4.9% from the total cohort). Second, several pre-analytical factors might affect the levels of CSF KFLCs and thus the KFLC index (5), e.g., late-onset (>50 years) progressive MS (37), renal dysfunction (38), blood contamination of CSF (39), storage or treatment of samples (40), and several therapies used to treat relapses, such as corticosteroids, intravenous immunoglobulins, or plasmapheresis (40). Furthermore, highly-effective DMTs might also decrease levels of the KFLC index (41), unlike other therapies (10, 42). Thus, we ensured that none of our patients had renal impairment at the time of LP, and we excluded those previously treated with corticosteroids or DMTs. Third, this algorithm has limitations, with the most important one being the selection of patients with a CIS, as screening many patients with a very low probability of converting to MS would decrease the advantage of eliminating OCGB studies. On the other hand, a careful selection of cases to assay would be associated with a higher advantage of the proposed method combination.

In conclusion, we present a diagnostic algorithm that includes, first, a KFLC index quantification in patients with a CIS suggestive of MS. A diagnosis of MS can be established in cases with a positive result, with additional OCGB assessment required for patients with negative KFLC index results. Therefore, we suggest considering both OCGBs and the KFLC index for demonstrating intrathecal IgG synthesis when applying the McDonald criteria for diagnosing MS.

## Data availability statement

The raw data supporting the conclusions of this article will be made available by the authors, without undue reservation upon reasonable request.

## Ethics statement

The studies involving humans were approved by Comité de Ética de la Investigación del Hospital Universitario Ramón y Cajal. The studies were conducted in accordance with the local legislation and institutional requirements. The participants provided their written informed consent to participate in this study.

## Author contributions

EM: Conceptualization, Formal Analysis, Investigation, Methodology, Writing – original draft, Writing – review & editing. JF-V: Conceptualization, Data curation, Investigation, Methodology, Resources, Writing – original draft, Writing – review & editing. AG-S: Investigation, Methodology, Writing – review & editing. SS: Investigation, Resources, Writing – review & editing. ME: Investigation, Methodology, Resources, Writing – review & editing. NV: Funding acquisition, Investigation, Methodology, Resources, Writing – review & editing. FR-J: Data curation, Investigation, Writing – review & editing. JC-G: Data curation, Investigation, Writing – review & editing. RS-A: Investigation, Writing – review & editing. JM: Investigation, Project administration, Validation, Visualization, Writing – review & editing. LC-F: Data curation, Investigation, Resources, Writing – review & editing. LV: Conceptualization, Funding acquisition, Investigation, Methodology, Project administration, Supervision, Validation, Visualization, Writing – original draft, Writing – review & editing.
