# Supplementary material for: Establishing the best combination of the kappa free light chain index and oligoclonal bands for an accurate diagnosis of multiple sclerosis
Source: Front Immunol. 2023 Oct 25;14:1288169. doi: 10.3389/fimmu.2023.1288169 (PMC10634415; doi:10.3389/fimmu.2023.1288169)
Supplement: Supplementary file 1 [file DataSheet_1.docx]

Supplementary Material

# Supplementary Tables

| **Supplementary Table 1. Baseline differences between patients with MS stratified by KFLC index above and below 6.1 (n = 320)** | | | |
| --- | --- | --- | --- |
|  | KFLC <6.1  (n = 54) | KFLC ≥6.1  (n = 276) | P value |
| Female | 31 (70.5) | 191 (69.2) | 0.87 |
| Age at LP (years) | 37.7 (30.7 – 43.5) | 33.8 (27.2 – 43.5) | 0.11 |
| Time to LP after first relapse (months) | 3.92 (1.26 – 9.79) | 4.05 (1.21 – 8.03) | 0.74 |
| Topography of first relapse |  |  |  |
| Optic nerve | 6 (13.6) | 55 (19.9) | 0.08 |
| Brainstem | 14 (31.8) | 59 (21.4) |  |
| Spinal cord | 13 (29.6) | 121 (43.8) |  |
| Multifocal | 1 (2.27) | 7 (2.5) |  |
| Cerebral hemisphere | 10 (22.7) | 29 (10.5) |  |
| Paroxysmal symptoms | 0 | 5 (1.8) |  |
| EDSS score at baseline | 1.5 (1.5 – 2) | 1.5 (1 – 2) | 0.32 |
| T2 lesions at baseline |  |  |  |
| 0 | 1 (2.27) | 10 (3.6) | 0.10 |
| 1 – 3 | 9 (20.5) | 32 (11.6) |  |
| 4 – 9 | 17 (38.6) | 75 (27.2) |  |
| 10 – 50 | 16 (36.4) | 132 (47.8) |  |
| >50 | 1 (2.2.7) | 27 (9.8) |  |
| Patients with enhancing lesions | 20/37 (54.1) | 142/245 (57.9) | 0.65 |
| DMT use during follow-up | 26 (59.1) | 229 (83.0) | <0.001 |
| CSF KFLC levels | 0.33 (0.27 – 0.45) | 3.33 (1.28 – 7.63) | <0.001 |
| Serum KFLC levels | 15.7 (12.7 – 18.5) | 14.4 (12.2 – 17.4) | 0.09 |
| Time of follow-up (years) | 7.21 (4.43 – 9.54) | 6.34 (3.99 – 10.9) | 0.41 |

Abbreviations: DMT: disease-modifying treatments; EDSS: expanded disability status scale; KFLC: kappa free light chain; LP: lumbar puncture.
Categorical variables are shown as number (%). Continuous variables are described as median (interquartile range).

| **Supplementary Table 2. Diagnostic value of OCGB and KFLC index ≥6.1 according to fulfillment of DIS and/or DIT criteria for MS at baseline MRI** | | | | |
| --- | --- | --- | --- | --- |
| Baseline MRI criteria | OCGB | | KFLC index ≥6.1 | |
|  | Positive  (n = 311) | Negative  (n = 60) | Positive  (n = 278) | Negative  (n = 93) |
| Neither DIS or DIT (n = 52) | 21 (40.4%) | 31 (59.6%) | 19 (36.5%) | 33 (63.5%) |
| DIS without DIT (n = 134) | 125 (93.3%)^a^ | 9 (6.7%) | 112 (83.6%)^a^ | 22 (16.4%) |
| DIS and DIT (n = 167) | 159 (95.2%)^a^ | 8 (4.8%) | 147 (88.0%)^a^ | 20 (12.0%) |

Abbreviations: DIS: dissemination in space; DIT: dissemination in time; KFLC: kappa free light chain; MS: multiple sclerosis; OCGB: oligoclonal IgG bands.

^a^P < 0.05 for comparison between OCGB and KFLC index using the McNemar test.

# Supplementary Figures


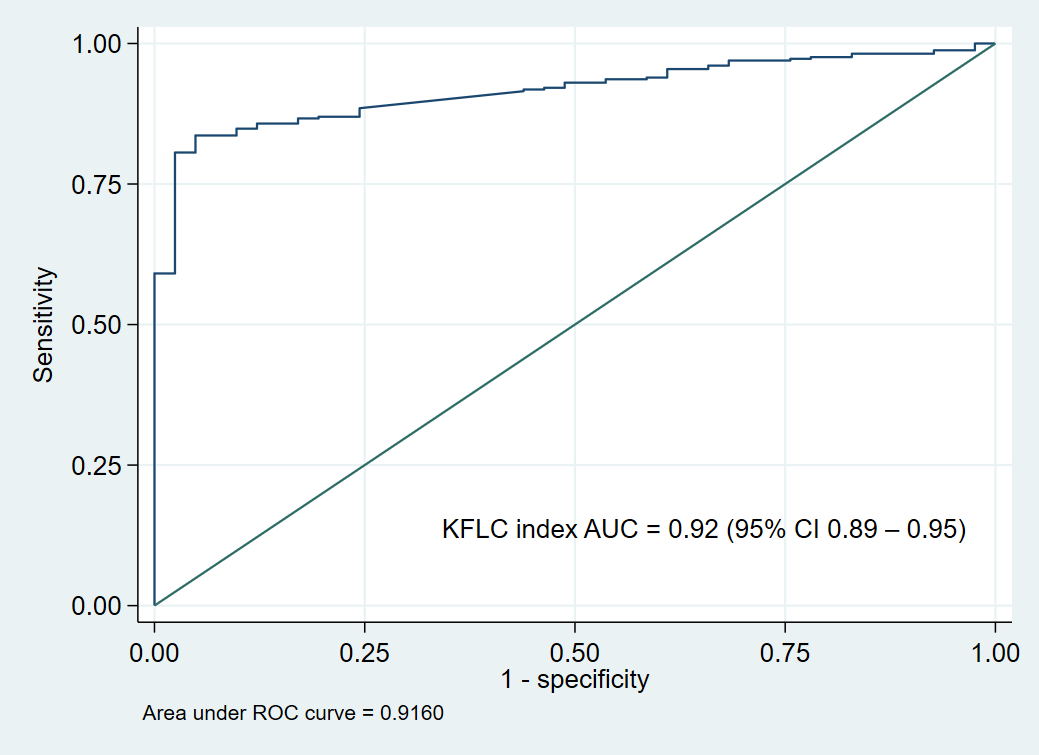


**Supplementary Figure 1.** ROC curve of KFLC index for the discrimination between MS and CIS. Abbreviations: CIS: clinically isolated syndrome; KFLC: kappa free light chain; MS: multiple sclerosis; ROC: receiver operating characteristic.


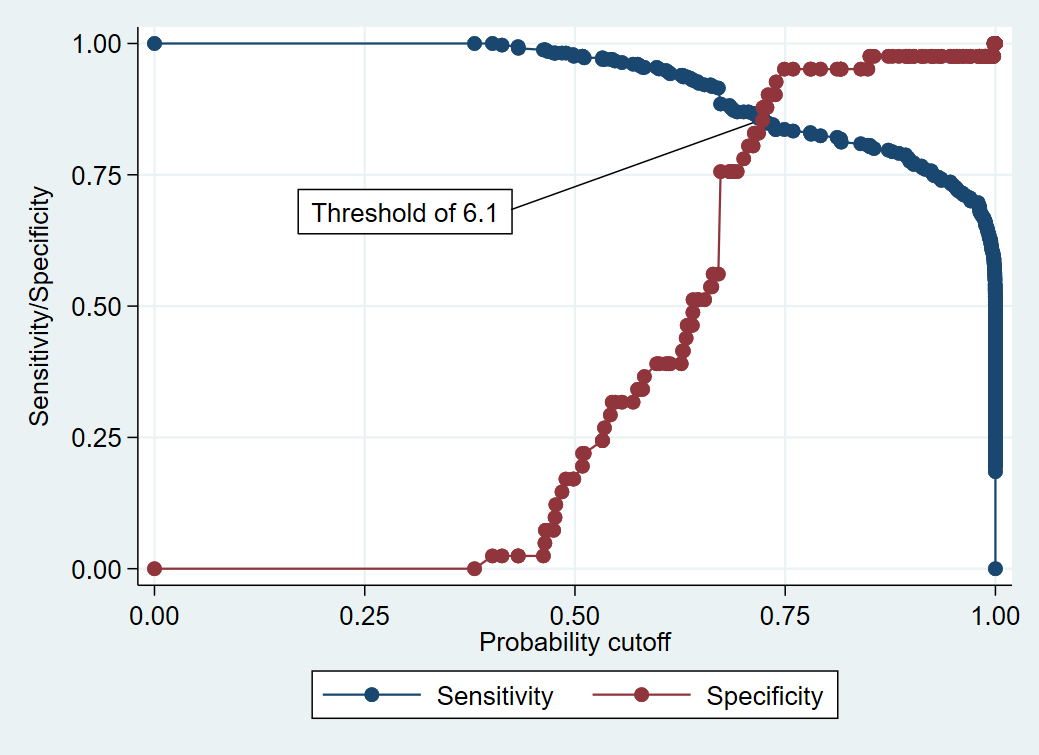


**Supplementary Figure 2.** Calculation of the best KFLC index cut-off value to discriminate patients with MS. Based on the Youden index, the KFLC index cut-off value of 6.1 showed the highest sensitivity (86.3%, 95% CI 82.7 – 89.8) and specificity (93.9%, 95% CI 91.5 – 96.4). Abbreviations: CI: confidence interval; KFLC: kappa free light chain.
